# A Comparison of the Flavonoid Biosynthesis Mechanisms of *Dendrobium* Species by Analyzing the Transcriptome and Metabolome

**DOI:** 10.3390/ijms231911980

**Published:** 2022-10-09

**Authors:** Sian Liu, Hanyue Zhang, Yingdan Yuan

**Affiliations:** College of Horticulture and Plant Protection, Yangzhou University, Yangzhou 225009, China

**Keywords:** *Dendrobium*, flavonoids biosynthesis, transcriptome, metabolome

## Abstract

*Dendrobium huoshanense*, *Dendrobium officinale*, and *Dendrobium moniliforme*, as precious Chinese medicinal materials, have a variety of medicinal properties. Flavonoids are important medicinal components of *Dendrobium*, but their accumulation rules and biosynthesis mechanisms remain unclear. To explore the similarities and differences of flavonoid accumulation and biosynthesis in these three *Dendrobium* species, we performed flavonoid content determination, widely-targeted metabolomics and transcriptome sequencing on 1–4 years old *Dendrobium* species. The results showed that in different growth years, *D. huoshanense* stems had the highest flavonoid content in the second year of growth, while *D. officinale* and *D. moniliforme* stems had the highest flavonoid content in the third year of growth. A total of 644 differentially accumulated metabolites (DAMs) and 10,426 differentially expressed genes (DEGs) were identified by metabolomic and transcriptomic analysis. It was found that DAMs and DEGs were not only enriched in the general pathway of “flavonoid biosynthesis”, but also in multiple sub-pathways such as “Flavone biosynthesis”, and “Flavonol biosynthesis” and “Isoflavonoid biosynthesis”. According to a combined transcriptome and metabolome analysis, the expression levels of the *F3′H* gene (*LOC110096779*) and two *F3′5′H* genes (*LOC110101765* and *LOC110103762*) may be the main genes responsible for the differences in flavonoid accumulation. As a result of this study, we have not only determined the optimal harvesting period for three Dendrobium plants, but also identified the key genes involved in flavonoid biosynthesis and provided a basis for further study of the molecular mechanism of flavonoid synthesis.

## 1. Introduction

*Dendrobium* is the second largest genus of Orchidaceae and is mainly distributed in tropical and subtropical regions [1,2]. There are more than 1500 species of *Dendrobium* in the world at present [3,4]. *Dendrobium* contains flavonoids, polysaccharides, alkaloids, phenanthrene, bibenzyl and sesquiterpenes, as well as other biologically active compounds [5,6]. Studies have shown that the stem of *Dendrobium* contains more medicinal components than other parts, and the Chinese Pharmacopoeia also specifies that the stem is the medicinal tissue [7,8,9]. Additionally, *Dendrobium* species differ in their chemical composition, content, pharmacology, and commercial value. *D. huoshanense, D. officinale, and D. moniliforme* are three species of *Dendrobium* with important medicinal value. Among them, *D. huoshanense* and *D. officinale* have been listed in the Chinese Pharmacopoeia, which indicates their high commercial value. In contrast, due to less research being completed on *D. moniliforme*, its medicinal value has not received enough attention, so it has not been listed in the Chinese Pharmacopoeia, resulting in its low commercial value. However, recent studies suggest that the medicinal components in *D. moniliforme* have antioxidant, anti-inflammatory, and therapeutic properties for osteoporosis [10,11]. As a result of these findings, it appears to be worthwhile to investigate the medicinal value of *D. moniliforme*, as well as *D. huoshanense* and *D. officinale*.

Studies on *Dendrobium* mainly focus on the isolation and identification of active components [12], genome analysis [13], biological activity [14,15], rhizosphere microbial composition [16], and pharmacological effects [11]. In recent years, research on the biosynthesis and accumulation mechanism of bioactive components of *Dendrobium* has also been carried out, but mainly focused on alkaloids and polysaccharides [17,18,19]. As one of the main bioactive components of *Dendrobium*, flavonoids have various beneficial effects on the human body, such as anti-cancer [20], antioxidant [21], promoting cognitive function [22], protecting nerves [23], and improving vascular function [24]. However, the biosynthesis and regulatory mechanisms of flavonoids in *Dendrobium* are still unclear. Therefore, it is necessary to know whether there are similarities and differences between different *Dendrobium* species in terms of flavonoid content and the biosynthesis pathway, which will be helpful for a comprehensive evaluation of the different *Dendrobium* species.

The accumulation of flavonoids has an age-dependent effect. In the leaves of 1–7-year-old ginkgo young trees, the flavonoid content decreased with increasing tree age, while the flavonoid content in the leaves of mature trees (ginkgo trees older than 25 years) did not change significantly [25,26]. However, the main medicinal tissue of *Dendrobium* is the stems, and the accumulation rule and regulation mechanism of flavonoids in the stems of *Dendrobium* are still unclear. Therefore, to explore the similarities and differences in the accumulation rules and regulatory mechanisms of the three *Dendrobium* flavonoids, we performed transcriptome and metabolome analysis on the stems of *D. huoshanense*, *D. officinale*, and *D. moniliforme* of 1–4 years old. We identified the dynamics of flavonoid accumulation in three *Dendrobium* species during different growth years as well as the molecular mechanism involved in flavonoid biosynthesis. WGCNA analysis was also used to screen out genes that are highly related to flavonoid synthesis. This study evaluated the similarity and specificity of flavonoid content and biosynthesis pathway of three *Dendrobium* species in order to discern the differences between them, which is of great significance for the comprehensive evaluation of three *Dendrobium* species, as well as providing theoretical support for the selection of a reasonable harvest period.

## 2. Results

### 2.1. Determination of Flavonoids and Alkaloids Contents

**Figure 1:**
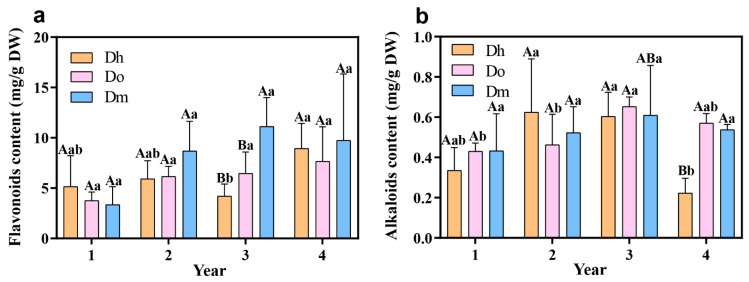
Determination of total flavonoids and alkaloids in stems of three *Dendrobium* species. (**a**) total flavonoids content in stems of three *Dendrobium* species of four growth years. (**b**) Total alkaloid content in stems of three *Dendrobium* species of four growth years. Capital letters indicate differences among different species and lowercase letters indicate differences among different growth years.

We determined the contents of total flavonoids and alkaloids in *D. huoshanense*, *D. officinale*, and *D. moniliforme* of different growth years. There was no consistency between the variation trends of the three *Dendrobium* species during different growth years. As for flavonoids, there were no significant differences between the three *Dendrobium* species in the first and fourth years of cultivation, but in the second and third years, *D. moniliforme* had significantly higher flavonoid levels than *D. huoshanense* and *D. officinale* (*p* < 0.05, Figure 1a). For *D. huoshanense*, the content of flavonoids in the third year was significantly different from that in other years (*p* < 0.05, Figure 1a). Flavonoids were highest in three-year-old *D. moniliforme* (11.13 mg·g^−1^), and lowest in one-year-old *D. moniliforme* (3.35 mg·g^−1^) (Figure 1a). Alkaloids were not significantly different between the three *Dendrobium* species in the first two years, but the total alkaloids content of *D. huoshanense* was significantly lower than that of *D. moniliforme* and *D. officinale* in the fourth year (Figure 1b). For *D. huoshanense*, the content of alkaloids in the fourth year was significantly different from that in other years (*p* < 0.05, Figure 1b). Three-year-old *D. officinale* contained the highest amount of alkaloid (0.65 mg·g^−1^), while four-year-old *D. huoshanense* contained the lowest amount (0.22 mg·g^−1^) (Figure 1b). Interestingly, the total flavonoids and alkaloids in *D. moniliforme* followed the same trend, namely, that they gradually increased from 1Dm to 3Dm and decreased from 3Dm to 4Dm, indicating that the medicinal value of *D. moniliforme* was at its highest during the third year of growth (Figure 1a,b).

### 2.2. Identification of DAMs and DEGs

**Figure 2:**
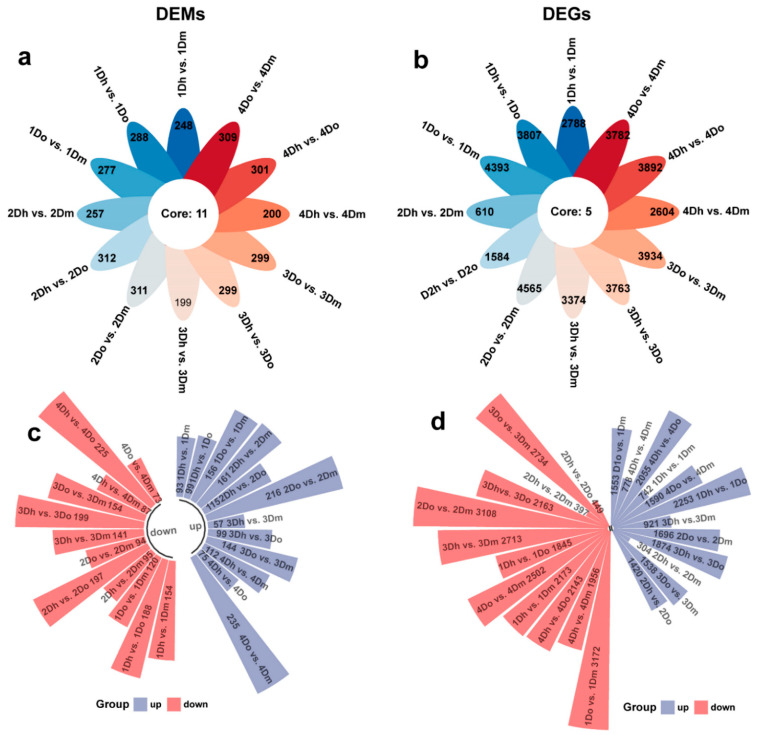
Changes in the number of DAMs and DEGs in 12 comparison groups. The total number of DAMs (**a**) and DEGs (**b**) in different comparison groups is calculated. The number in the center represents the number of DAMs/DEGs shared by all comparison groups. The number of DAMs (**c**) and DEGs (**d**) that are upregulated and downregulated in different comparison groups is compared. Blue represents downregulation and red represents upregulation.

The clean reads obtained from the transcriptome sequencing of *D. huoshanense*, *D. officinale*, and *D. moniliforme* were mapped to the *D. officinale* genome. The results showed that these clean reads all had high mapping rates and could be used for subsequent comparative transcriptome analysis (Table S1). In order to further reveal the differences of transcriptome in *D. huoshanense*, *D. officinale*, and *D. moniliforme* under different growth years, we constructed 12 comparison groups: 1Dh vs. 1Dm; 1Dh vs. 1Do; 1Do vs. 1Dm; 2Dh vs. 2Dm; 2Dh vs. 2Do; 2Do vs. 2Dm; 3Dh vs. 3Dm; 3Dh vs. 3Do; 3Do vs. 3Dm; 4Dh vs. 4Dm; 4Dh vs. 4Do; 4Do vs. 4Dm. Based on the *p*-value < 0.05 and | log2 (FC) | > 1 for the threshold, we screened 644 DAMs and 10426 DEGs (Figure 2a,b).

In terms of DAMs, the number of DAMs detected in 2Do vs. 2Dm was the highest (310), of which 216 DAMs were upregulated and 94 DAMs were downregulated. The number of DAMs detected in 4Dh vs. 4Dm was the lowest (199), among which 112 DEGs were upregulated and 87 DEGs were downregulated. In addition, the proportion of upregulated metabolites was larger in 1Do vs. 1Dm, 2Dh vs. 2Dm, 2Do vs. 2Dm, 4Dh vs. 4Dm, and 4Do vs. 4Dm, and the proportion of downregulated metabolites was greater in other comparison groups (Figure 2c). In terms of DEGs, the highest number (4565) was detected in 2Do vs. 2Dm, with 1665 DEGs that were upregulated and 2900 that were downregulated. The lowest number of DAMs was detected in 2Dh vs. 2Dm (609), with 264 upregulated DEGs and 345 downregulated DEGs. In addition, the proportion of upregulated genes was larger in 1Dh vs. 1Do and 2Dh vs. 2Do, and the proportion of downregulated genes was larger in other comparison groups (Figure 2d).

### 2.3. KEGG Enrichment of DAMs

**Figure 3:**
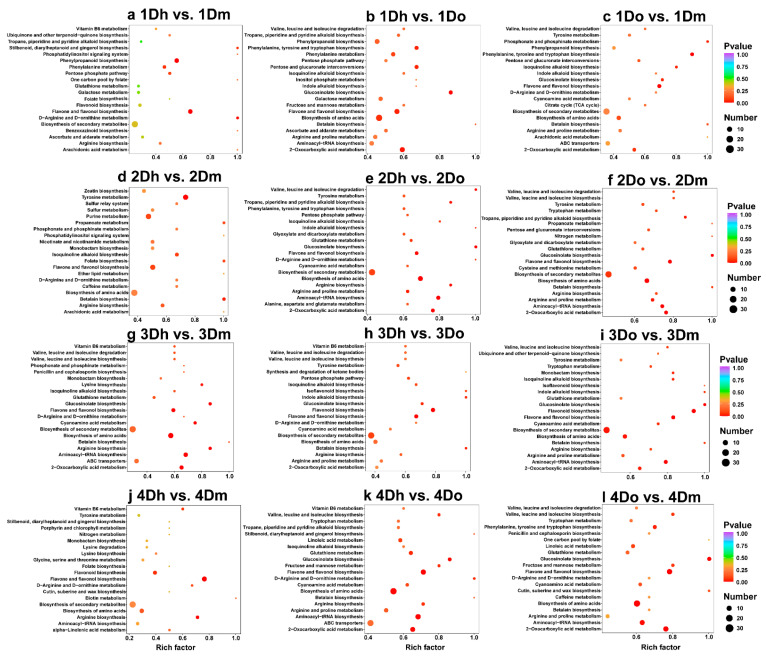
KEGG enrichment analysis of DAMs. The X-axis label represents the rich factor, and the Y-axis label shows the KEGG pathways. Colors indicate *p*-values (from the lowest in red to the highest in blue), red indicates significant enrichment (*p* < 0.05), and green indicates not significantly enriched. The size of the bubbles represents the number of DAMs. The rich factor represents the ratio of the number of DAMs in each pathway to the total metabolites, and the larger the rich factor, the higher the enrichment of the pathway.

To further reveal the potential role of DAMs, KEGG enrichment analysis was performed (Figure 3a–l). The pathway of significant enrichment may be the key to reveal the changes of metabolites of the three *Dendrobium* species in different growth years. The results showed that the pathways associated with flavonoid biosynthesis were enriched. Among all of the comparison groups, “Flavone and flavonol biosynthesis” was enriched in all of the comparison groups (Figure 3a–l). “Flavonoid biosynthesis” was enriched in 3Do vs. 3Dm, 4Dh vs. 4Dm and 4Dh vs. 4Do (Figure 3i–k), and “Isoflavonoid biosynthesis” was enriched in 3Do vs. 3Dm (Figure 3i). It is noteworthy that the phenylalanine-related pathway was the main enrichment pathway in the three comparison groups of annual *Dendrobium*, such as “Phenylpropanoid biosynthesis”, “Phenylalanine metabolism”, and “Phenylalanine, tyrosine and tryptophan biosynthesis” (Figure 3a–c).

Additionally, the alkaloid-related pathways were enriched. “Tropane, piperidine and pyridine alkaloid biosynthesis” was enriched in five comparison groups (Figure 3a,b,e,f,k). “Isoquinoline alkaloid biosynthesis” was enriched in eight comparison groups. Polysaccharide-related pathways were also obviously enriched (Figure 3b–e,g–i,k). For example, “Galactose metabolism” was enriched in 1Dh vs. 1Dm and 1Dh vs. 1Do (Figure 3a,b), and “Fructose and mannose metabolism” was enriched in 1Dh vs. 1Do (Figure 3b). In addition, “Aminoacyl-tRNA biosynthesis”, “2-Oxocarboxylic acid metabolism” and “ABC transporters” were also significantly enriched in multiple comparison groups.

### 2.4. GO Enrichment of DEGs

**Figure 4:**
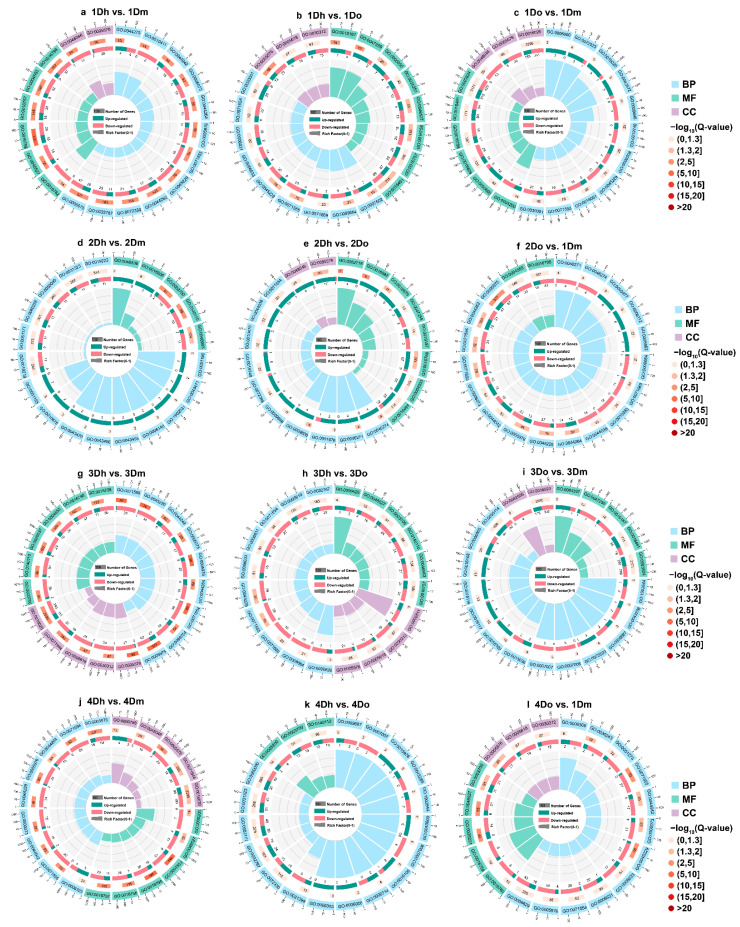
GO enrichment analysis of DEGs. The secondary categories of GO include Biological Process (BP), Molecular Function (MF), and Cellular Component (CC). These figures consist of four circles from outside to inside. The first circle is the enriched classification, and the outer circle is the scale of the number of genes. The combination of letters and numbers is the ID of the GO term. The three colors represent BP, MF, and CC, respectively. The second circle is the number of background genes and the q-value of the GO term. The *p*-value after FDR correction is called q-value. The more DEGs are enriched, the longer the box is. The smaller the q-value, the darker the color of the box, indicating the higher the degree of enrichment. The third circle represents the number of up- and downregulated genes. Red indicates upregulated expression, green indicates downregulated expression, and the numbers under the box represent the number of upregulated or downregulated genes. The fourth circle is the rich factor of each GO term (the ratio of the number of DEGs in the GO term to the number of background genes), and each cell of the background auxiliary line represents 0.1.

In order to further study the function of DEGs in the stems of three *Dendrobium* species with different growth years, we annotated it into three categories based on the GO database, including biological process (BP), cell component (CC), and molecular function (MF). In the top 20 significantly enriched GO terms of all of the comparison groups, the proportion of BP class was the highest, and CC class was the lowest. Except for 2Dh vs. 2Dm and 2Dh vs. 2Do, the number of downregulated DEGs was higher in other comparison groups (Figure 4).

In the top 20 significantly enriched GO terms, 1Dh vs. 1Do and 3Dh vs. 3Do were significantly different from other comparison groups. In 1Dh vs. 1Do, there were nine GO terms enriched in the BP category, among which “Plant-type cell wall organization” was enriched the most (Figure 4b), and the number of DEGs enriched in “Cell wall organization or biogenesis” was the most. There are eight GO items enriched in the MF category, among which the number of DEGs enriched in “Oxidoreductase activity” was significantly more than other GO terms (Figure 4b). In 3Dh vs. 3Do, the BP, CC and MF categories were enriched in 10, 4, and 6 GO terms, mainly including “Monocarboxylic acid metabolic process”, “Lipid biosynthetic process” and “Transferase activity, transferring glycosyl groups” (Figure 4h).

In addition, significantly enriched GO terms were similar in the other 10 comparison groups except 1Dh vs. 1Do and 3Dh vs. 3Do (Figure 4a,c–g,i–l). In the BP category, the GO terms were mainly enriched in metabolic process and catabolic process, including “Secondary metabolic processes”, “Phenylpropanoid metabolic process”, “Fructose metabolic process”, “Starch metabolic processes”, “Cellular carbohydrate catabolic process”, and “Lignin catabolic process” (Figure 4a,c–g,i–l). In the MF category, the GO terms were mainly enriched in transferase activity and hydrolase activity, including “Transferase activity, transferring hexosyl groups”, “Transferase activity, transferring glycosyl groups”, “Hydrolase activity, hydrolyzing O-glycosyl compounds”, “Hydrolase activity, acting on glycosyl bonds”. In the CC category, GO terms were mainly enriched in “Extracellular region” and “Apoplast” (Figure 4a,c–g,i–l). In general, DEGs were significantly enriched in the metabolic process in the top 20 enriched GO terms, which further indicated that the metabolic process played an important and dominant role in the growth of the three *Dendrobium* species.

### 2.5. KEGG Enrichment of DEGs

**Figure 5:**
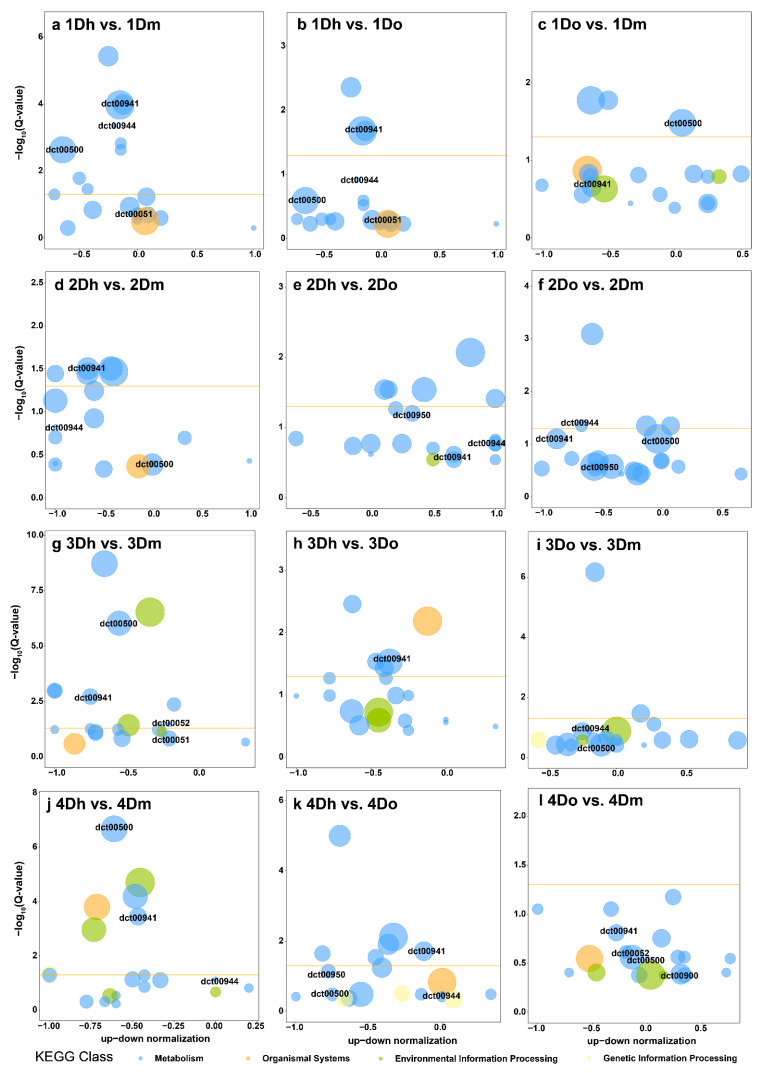
KEGG enrichment bubble diagram of DEGs. X-axis represents Z-score. Y-axis represents-log_10_ (*p*-value). Bubble size represents the number of DEGs, and bubble color represents KEGG classification. The orange threshold line indicates *p* = 0.05.

In each comparison group, the DEGs located at “Metabolism” accounted for the largest proportion of enrichment pathways, followed by “Environmental Information Processing” and “Genetic Information Processing”. There were 12 comparison groups and almost all of them involved pathways related to flavonoid biosynthesis. “Phenylpropanoid biosynthesis” was significantly enriched in the comparison groups except 2Do vs. 2Dm, 3Do vs. 3Dm, and 4Do vs. 4Dm (Figure 5f,i,l). “Flavone and flavonol biosynthesis” was significantly enriched in 1Dh vs. 1Dm, 2Do vs. 2Dm, and 3Dh vs. 3Dm (Figure 5a,f,g). “Flavonoid biosynthesis” was significantly enriched in all of the comparison groups except 2Dh vs. 2Do, 3Do vs. 3Dm, and 4Do vs. 4Dm (Figure 5a–d,f–h,j,k).

In addition, we found that the pathways related to polysaccharides, alkaloids, fatty acids, phenylalanine, and other metabolites were significantly enriched. For example, “Starch and sucrose metabolism” was significantly enriched in 1Dh vs. 1Dm, 1Do vs. 1Dm, 3Dh vs. 3Dm, and 4Dh vs. 4Dm (Figure 5a,c,g,j). “Tropane, Piperidine and Pyridine alkaloid biosynthesis” was significantly enriched in 2Dh vs. 2Dm (Figure 5d). “Fatty acid elongation” was significantly enriched in all of the comparison groups except 2Dh vs. 2Dm, 4Dh vs. 4Dm and 4Do vs. 4Dm (Figure 5a–c,e–i,k). “Phenylalanine metabolism” was significantly enriched in 2Dh vs. 2Dm and 4Dh vs. 4Do (Figure 5d,k). In summary, DEGs were largely enriched in metabolite-related pathways, suggesting that they may participate in multiple metabolic processes and play multiple biological roles.

### 2.6. Integrative Analysis of DAMs and DEGs

**Figure 6:**
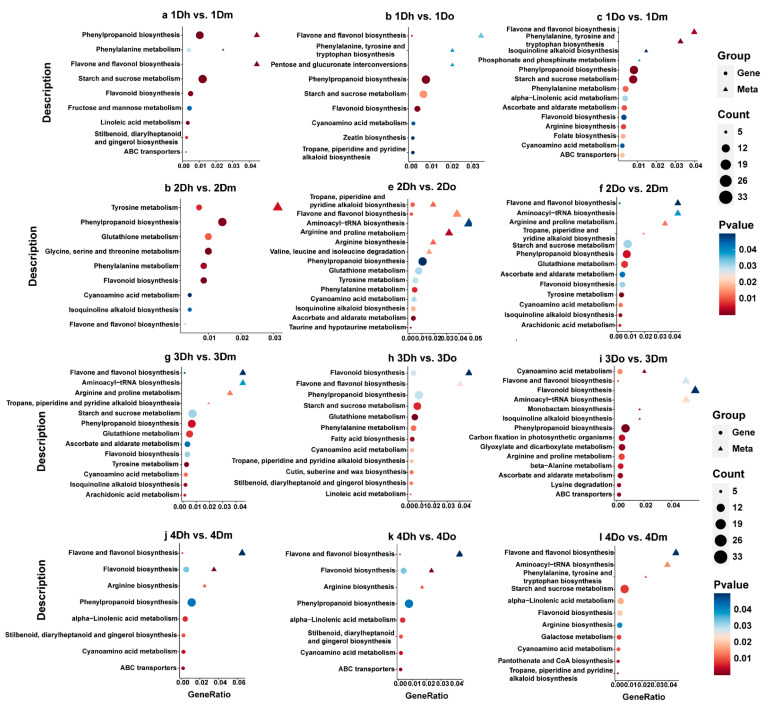
KEGG enrichment analysis of DAMs and DEGs. The color of the points indicates the degree of enrichment and the size of the points indicates the number of enriched DAMs or DEGs. The circle represents DEGs and the triangle represents DAMs.

The DEGs and DAMs in each comparison group were mapped to KEGG pathways to analyze the relationship between metabolites and genes in three *Dendrobium* species. DAMs and DEGs were found to be closely related to the biosynthesis of flavonoids. The biosynthesis of phenylpropanoid, flavones and flavonols, and flavonoid was significantly enriched in almost all of the comparison groups. It is noteworthy that, in 1Dh vs. 1Dm, the DAMs and DEGs were simultaneously enriched to “Phenylpropanoid biosynthesis” (Figure 6a). In 1Dh vs. 1Do (Figure 6b), 2Dh vs. 2Do (Figure 6e), 2Do vs. 2Dm (Figure 6f), 3Dh vs. 3Dm (Figure 6g), 3Do vs. 3Dm (Figure 6i), 4Dh vs. 4Dm (Figure 6j), and 4Dh vs. 4Do (Figure 6k), the DAMs and DEGs were simultaneously enriched to “Flavone and flavonol biosynthesis”. In 3Dh vs. 3Do (Figure 6h), 4Dh vs. 4Dm (Figure 6j), and 4Dh vs. 4Do (Figure 6k), the DAMs and DEGs were simultaneously enriched to “Flavonoid biosynthesis”. These comparison groups showed significant differences in genes and metabolites related to flavonoid biosynthesis (Figure 6a–l).

Additionally, DAMs and DEGs were also significantly enriched in other metabolic processes besides the flavonoid synthesis pathway. For example, DAMs and DEGs were simultaneously enriched in “Tropane, Piperidine and Pyridine alkaloid biosynthesis” in 2Dh vs. 2Do (Figure 6e). It is speculated that the genes and metabolites in the biennial *D. huoshanense* and *D. officinale* may significantly affect the alkaloid synthesis pathway. DAMs and DEGs were simultaneously enriched to “Tyrosine metabolism” in 2Dh vs. 2Dm and to “Cyanoamino acid metabolism” in 3Do vs. 3Dm (Figure 6b,i). Interestingly, the significantly enriched pathways in 4Dh vs. 4Dm and 4Dh vs. 4Do were identical, including “Flavone and flavonol biosynthesis”, “Flavonoid biosynthesis”, “Phenylpropanoid biosynthesis”, and “ABC transporters” (Figure 6j,k). The results showed that the biosynthesis of flavonoids might play an important role in the four-year-old *Dendrobium* species.

### 2.7. Changes of Genes and Metabolites in Regulatory Networks for Flavonoid Biosynthesis

**Figure 7:**
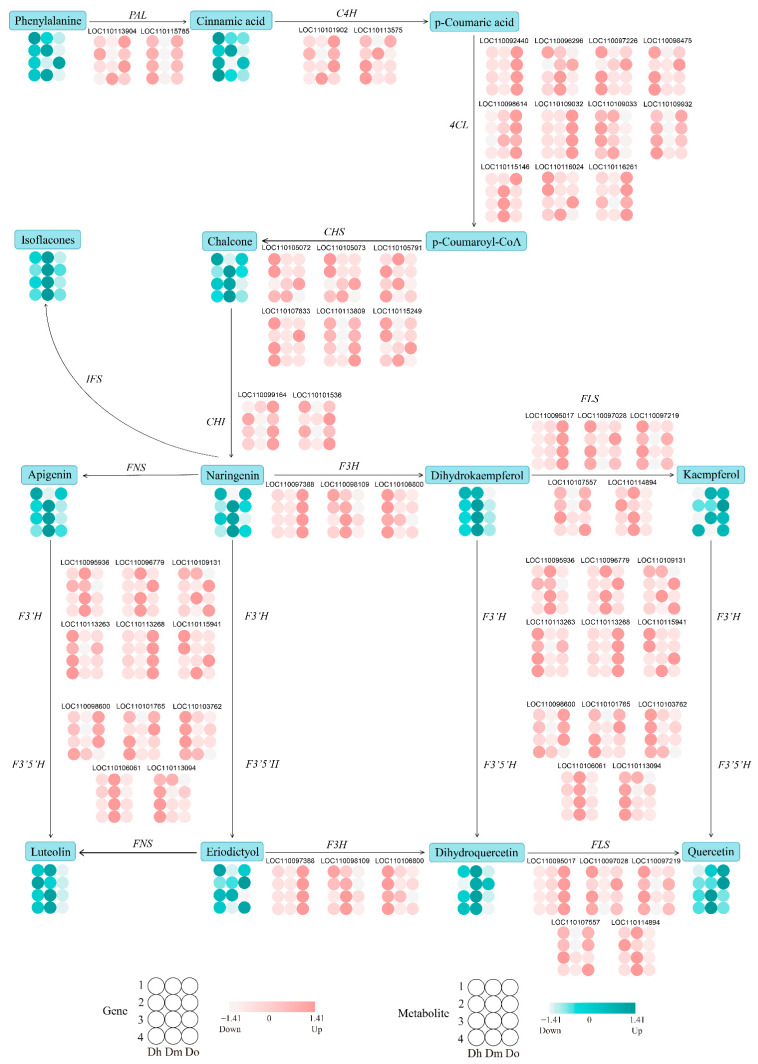
Flavonoid biosynthesis pathway of three *Dendrobium* species with different growth years. The four rows and three columns of circular patterns indicate the corresponding growth years and expression levels of Dendrobium species, respectively. Red represents DEGs and green represents DAMs.

We conducted a comprehensive analysis of transcript and metabolite levels associated with the flavonoid biosynthesis pathway in three *Dendrobium* species to better understand the relationship between genes and metabolites. Twelve DAMs of three *Dendrobium* species of different growth years were identified in the biosynthesis pathway of flavonoids, including phenylalanine, cinnamic acid, naringenin chalcone, naringenin, dihydroquercetin, quercetin, kaempferol, dihydrokaempferol, eriodictyol, apigenin, luteolin, and isoflavones (Figure 7). Additionally, we identified 42 DEGs that were involved in the synthesis of flavonoids (Figure 7).

When comparing the different growth years of the same *Dendrobium* specie, it was found that the concentration of flavonoids (naringenin chalcone, naringenin, kaempferol, eriodictyol, dihydroquercetin) and the expression levels of structural genes (*C4H*, *4CL*, *F3′H*, *F3′5′H*, *FLS*, etc.) in the flavonoid synthesis pathway were higher in the third and fourth years of cultivation in *D. huohanense*. Additionally, most of the flavonoids (naringenin chalcone, naringenin, apigenin, isoflavones, eriodictyol, dihydroquercetin, kaempferol) and the expression levels of structural genes (*PAL*, *C4H*, *4CL*, *CHS*, *F3H*, *F3′H*, *F3′5′H*, *FLS*) in the flavonoid synthesis pathway were higher in the third and fourth years of cultivation in *D. moniliforme*. However, most flavonoids (isoflavones, apigenin, kaempferol, quercetin, eriodictyol, dihydroquercetin, luteolin) and the expression levels of structural genes (*4CL*, *CHI*, *F3′H*, *F3′5′H*, *FLS*) in the flavonoid synthesis pathway were higher in the second year of cultivation in *D. officinale*. This suggests that the flavonoid synthesis genes may be the key to regulating flavonoid accumulation.

According to the results, most of the flavonoids (phenylalanine, cinnamic acid, naringenin Chalcone, dihydroquercetin, dihydrokaempferol, eriodictyol, apigenin, luteolin, isoflavones) and the expression levels of structural genes (*CHS*, *F3H*, *F3′H*, *F3′5′H*, *FLS*) were higher in in the first year of *D. huohanense*. In the second, third, and fourth years of cultivation, most of the flavonoids (naringenin chalcone, naringenin, dihydroquercetin, quercetin, dihydrokaempferol, apigenin, luteolin, isoflavones) were higher in *D. moniliforme*, but there were significant differences in gene expression levels among different *Dendrobium* species. In three *Dendrobium* species with different growth years, we found differences in the gene expression patterns and metabolite accumulation in the flavonoid biosynthesis pathway. The variation trends of most genes and their downstream metabolites were generally consistent.

### 2.8. Weighted Gene Co-Expression Network Analysis

**Figure 8:**
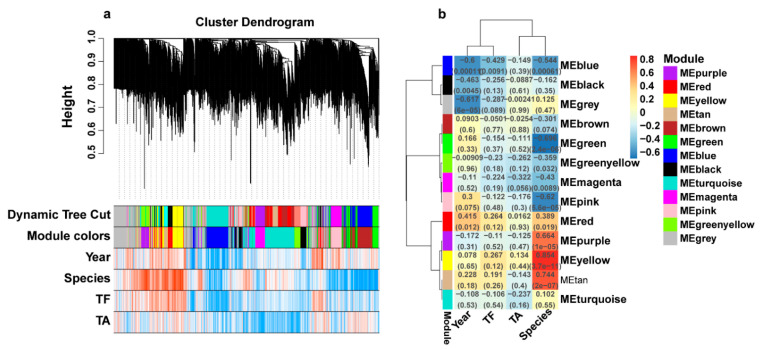
Co-expression network analysis of different growth years and different species. (**a**) Hierarchical clustering analysis tree of DEGs based on topological overlap. (**b**) The module-associated trait. Rows correspond to modules, and columns correspond to features. The number in each cell represents the corresponding correlation and *p*-value.

We conducted co-expression analysis and network construction in order to elucidate the relationship between the genes in different *Dendrobium* species of different growth years (Figure 8a,b). In total, 13 different modules were obtained, including purple, red, yellow, tan, brown, green, blue, black, turquoise, magenta, pink, green–yellow, and grey (Figure 8b). Furthermore, we examined the relationship between modules and traits (Year, TF, TA, species). Results showed that the blue and gray modules were significantly negatively correlated with Year; the blue, green, and pink modules were significantly negatively correlated with Species; and the purple, yellow, and tan modules were significantly positively correlated with Species. Based on these results, DEGs of these modules were closely correlated with the changes in *Dendrobium* species over time.

**Figure 9:**
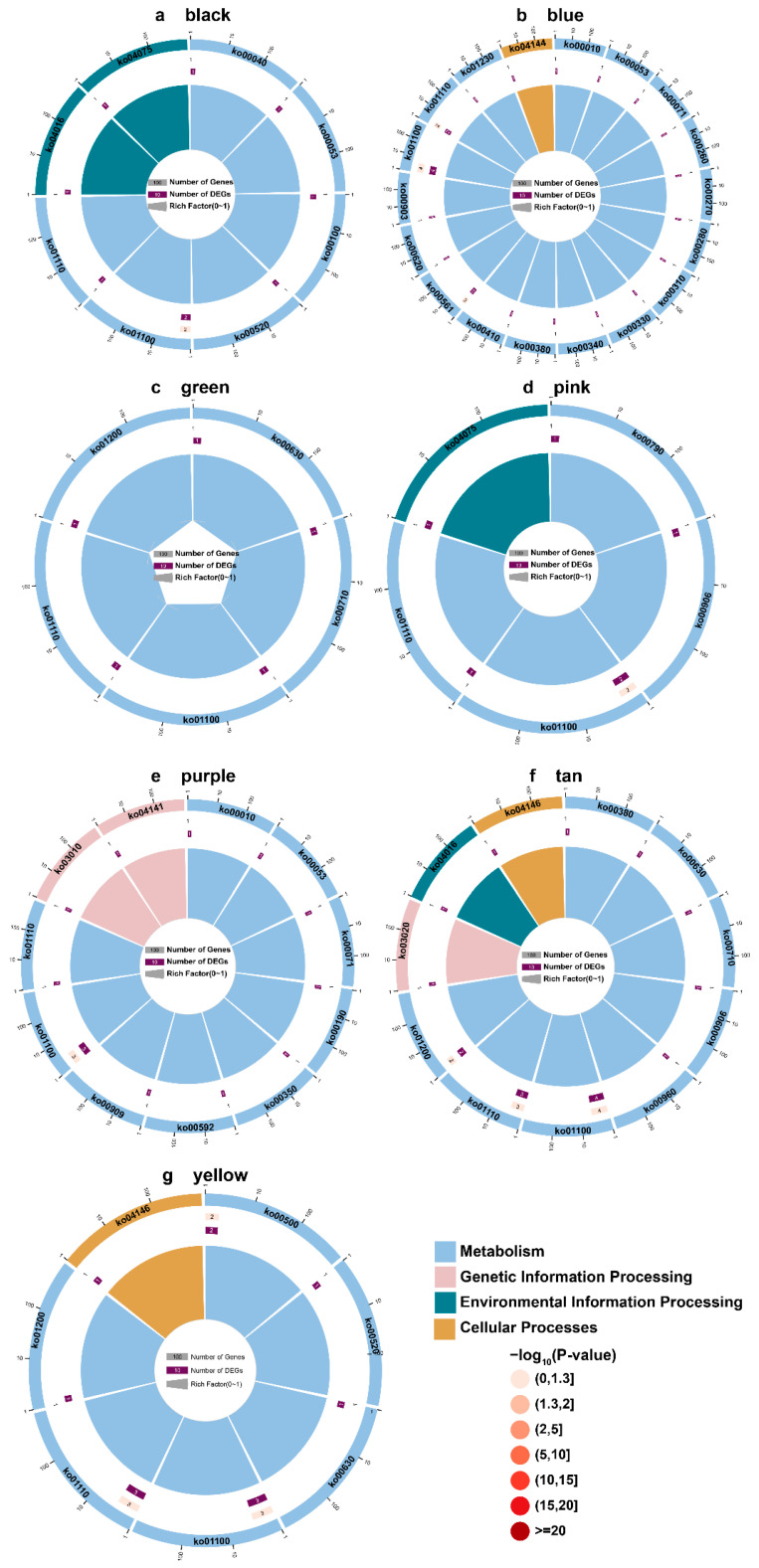
KEGG enrichment analysis of the 7 modules with high correlation. Different colors represent different categories.

As part of our investigation into the potential functions of genes in each module, we conducted KEGG enrichment analyses for genes in the black, blue, green, pink, purple, tan, and yellow modules (Figure 9a–g). The results showed that DEGs enriched to “Metabolism” were the most prevalent in all of the modules, while only a few DEGs were enriched to “Environmental information processing”, “Cellular processes”, and “Genetic information processing”. The term “metabolic pathways” was significantly enriched in all of the modules. In all of the modules except black, the term “biosynthesis of secondary metabolites” was significantly enriched (Figure 9b–g). As a result of these results, it can be concluded that metabolic processes have a dominant role in the growth of all three *Dendrobium* species. The enrichment of pathways related to polysaccharides and alkaloids was also noteworthy. For example, “Pentose and glucuronate interconversions” was significantly enriched in the black module (Figure 9a). “Glycolysis/Gluconeogenesis” was significantly enriched in the blue and purple module (Figure 9b,e). “Starch and sucrose metabolism” was significantly enriched in the yellow module (Figure 9g). “Sesquiterpenoid and triterpenoid biosynthesis” was significantly enriched in the purple module (Figure 9e). “Tropane, piperidine and pyridine alkaloid biosynthesis” was significantly enriched in the tan module (Figure 9f).

**Figure 10:**
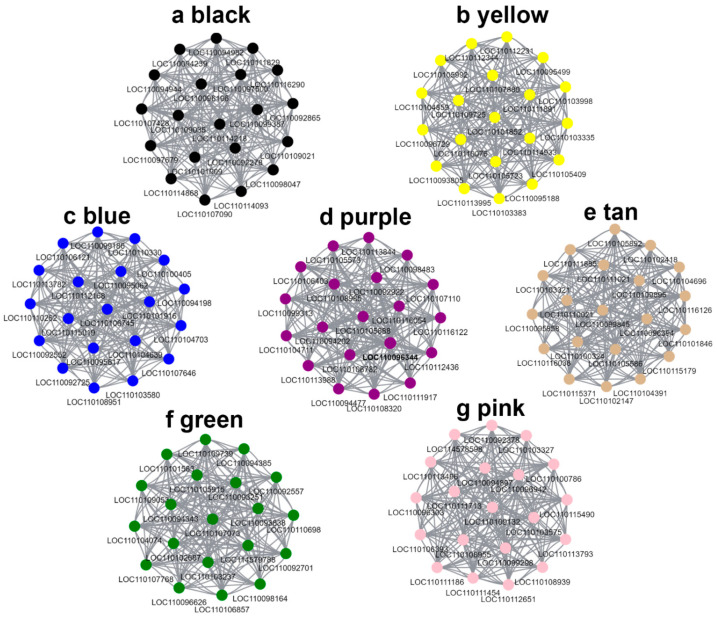
Network diagram of hub genes in 7 modules with high correlation.

In addition, we constructed seven gene-related networks using genes from these seven modules in order to identify key genes (Figure 10a–g). Based on their connectivity, the top 20 genes were considered Hub genes. In key pathways, the Hub gene is highly co-expressed with other genes. According to the results, these Hub genes were highly correlated and had a high degree of connectivity. Moreover, we found that most of the genes in the green, pink, purple, and tan modules were highly expressed in the three- and four-year-old *D. huohanense* (Figure 10d–g). In the three- and four-year-old *D. moniliforme*, most of the genes in the green, pink, and tan modules were highly expressed (Figure 10e–g). The majority of genes in the black, blue, and purple modules were highly expressed in the two-year-old *D. officinale* (Figure 10a,c,d). The expression pattern of the genes in the flavonoid synthesis pathway was consistent with this, suggesting that these modules might be closely related to flavonoid biosynthesis. In the green module, we identified a member of the bHLH transcription factor family bHLH143 (LOC110101583) and a member of the R2R3-MYB transcription factor family MYB6 (LOC110092557), which are known to regulate flavonoid biosynthesis (Figure 10f). The purple module also contained a gene LOC110105573 for sesquiterpene synthase (SES), which was involved in the synthesis of alkaloids (Figure 10d).

## 3. Discussion

Bioactive components have different accumulation patterns at different developmental stages and at different ages of plants. The total phenolic content of *Astragalus compactus* was higher at the fruit stage in comparison to the vegetative growth stage and flowering stage [27]. In the roots and root hairs of 1–5-year-old *Panax ginseng*, the content of ginsenosides and triterpenoids was positively correlated with age [28], while the contents of triterpenoids in the roots of 2, 3, 4, and 10 years old *Codonopsis lanceolata* were negatively correlated with age, and the carbohydrate content showed a trend of first increasing and then decreasing [29]. Polysaccharides, alkaloids, and flavonoids are the main active ingredients of *Dendrobium*. According to previous studies, there is a difference in the polysaccharide and alkaloid content among the three *Dendrobium* species [30]. By using UPLC and ICP-MS, Yuan et al. found that the three kinds of three-year-old *Dendrobium* species ranked in order of polysaccharide content as follows: *D. huoshanense* > *D. moniliforme* > *D. officinale* [30]. Based on the content of arabinose, rhamnose, xylose, and other monosaccharides, Chen et al. found the three species of wild and tissue-cultured *Dendrobium* arranged in the same order [31]. The total alkaloid content of three-year-old *D. huoshanense* was reported to be slightly higher than that of two other three-year-old *Dendrobium* species [30]. Although the content of total alkaloids was not significantly different among the three *Dendrobium* species grown at three years, it may be due to the fact that the plant materials were harvested at different times. Currently, most studies on *Dendrobium* have focused on polysaccharides and alkaloids in *D. officinale* [32,33], with few reports on comparing flavonoids among *D. huoshanense*, *D. moniliforme*, and *D. officinale* of different growth years. During the second and third years of cultivation, the total flavonoid content of *D. moniliforme* was significantly higher than that of *D. huoshanense* and *D. officinale*, especially in the third year. During the second, third, and fourth years of cultivation, metabolic data analysis showed that *D. moniliforme* had a higher concentration of major flavonoid compounds than *D. huoshanense* and *D. officinale*. The results of this study indicate that *D. moniliforme* contains a higher content of flavonoids than *D. huoshanense* or *D. officinale*. Flavonoids are influenced by a number of factors. There is evidence that flavonoids accumulate in specific stages and tissues [34]. There was a significant increase in flavonoid composition in the roots of *Scutellaria baicalensis* before flowering, but there was little change after flowering [35]. The accumulation of flavonoids was greatest in the growing leaves of *Eucommia ulmoides*, followed by old leaves [36]. It was observed that the content of luteolin increased and then decreased during the process of growing peanuts in *Lonicerae japonicae* [37]. Moreover, different flavonoid components within a plant can also exhibit significant variations in their content. In *Scutellaria baicalensis* roots, baicalin content increased gradually with growth and development, while baicalein content decreased gradually [38]. As the *Prunus mume* ripens, the total flavonol content decreases, while catechin content increases [39]. The content of flavonoids in the stems of different Dendrobium species was also found to vary based on metabolic data. Major flavonoid components were more abundant in the third or fourth year of cultivation in *D. huoshanense* and *D. moniliforme*. During the second year of cultivation, *D. officinale* contained significantly more flavonols than during the third and fourth years. These results indicate that flavonoids are influenced by different developmental stages and are subject to change as plants grow and develop. Flavonoids were found to be closely related to the regulation of key genes. The low expression of *FLS* and high expression of *DFR* in *Camellia sinensis* resulted in the accumulation of anthocyanins during the development of pink *Camellia sinensis* [40]. At 2, 3, and 4 months of growth, *Anoectochilus roxburghii* exhibited an increase in flavonoids, which was associated with significant upregulation of the *CHS* gene [41].

In order to screen key genes for flavonoid biosynthesis and regulation, comparative transcriptome analysis of three *Dendrobium* species is necessary, however only *D. officinale* has genomes, while *D. huoshanense* and *D. moniliforme* do not have reference genomes. We previously performed de novo transcriptome analysis on data from *D. huoshanense* and *D. moniliforme*, yielding 478,361 and 562,580 unigenes, respectively [7,8]. *D. huoshanense* and *D. moniliforme* are closely related to *D. officinale*, however genome sequencing showed that *D. officinale* has only 28,910 genes [4]. These results indicate that de novo transcriptome analysis based on Illumina short-read sequencing will yield a large number of transcript fragments, which are not as reliable as transcriptome analysis based on reference genomes. Previous studies have found that the *D. officinale* genome can be used to analyze the transcriptome data of *D. huoshanense* and *D. moniliforme* with a high mapping rate [42]. Similar results were obtained in this study. Therefore, it is feasible to use *D. officinale* as a reference sequence for comparative transcriptome analysis of three *Dendrobium* plants. An analysis of transcriptomic and metabolomic data demonstrated that high expression of one *F3′H* gene (*LOC110096779*) and two *F3′5′H* genes (*LOC110101765*, *LOC110103762*) induced the accumulation of downstream flavonoids (Luteolin, Eriodictyol, Dihydroquercetin, Quercetin). It has been found that high expression of *F3′H* or *F3′5′H* in *Camellia sinensis* [43], *Pericallis hybrida* [44], *Aconitum carmichaelii* [45], and other plants can promote the flavonoid biosynthesis. Additionally, the expression of F3′H gradually increased during the opening of *Dendrobium* ‘Sonia Earsakul’ flowers, and its silencing could block the formation of endogenous anthocyanins [46]. Similarly, a low expression of *F3′H* resulted in white petals in *Dendrobium* hybrids [47]. *F3′5′H* is also a key gene for flavonoid biosynthesis in *Dendrobium*. In *D. moniliforme*, *F3′5′H* controls perianths’ coloration by producing reddish purple anthocyanins [48].

The content and composition of secondary metabolites in medicinal plants determine the optimal harvest time, as well as the quality of the medicinal plants. Therefore, the harvest time of medicinal plants should be considered comprehensively in relation to the accumulation of active components and the growth stage of the plants. *Astragalus compactus* fruit stages have been shown to contain increased total phenolic content, which enhances the antioxidant activity of leaves [27]. The best harvest time for *Lonicerae japonicae* is believed to be determined by the chlorogenic acid and luteolin content of the slightly white alabastrum and whole white alabastrum stages [37]. In *Scutellaria baicalensis*, after three years of growth, the active components decrease, so it is determined that the ideal harvest time is between two and three years [49]. It appears that the harvest time of medicinal plants varies with species, and the dynamic change in the accumulation of active components is a significant indicator of the harvest time.

In recent studies, flavonols such as kaempferol, eriodictyol, and quercetin have shown a wide range of pharmacological effects, making them effective drugs for the prevention and treatment of various diseases [50]. Kaempferol, for example, is best known for its anti-inflammatory properties in the treatment of chronic and acute inflammation [51,52]. Quercetin has also been found to be effective in the prevention and treatment of cancer [53,54]. Eriodictyol has excellent therapeutic effects on antipyretic analgesia, heart protection, diabetes prevention, immune regulation, and other areas [55]. Flavonoids are widely found in orchidaceae, and flavone C-glycosides and flavonols are the most common components [56]. More than 89 flavonoids have been found in *Dendrobium*, naringenin, quercetin, and rutin are widely present and at a high content level [57]. These components have pharmacological activities such as antioxidant, hypoglycemic, can improve blood circulation, lower cholesterol, and have a protective effect on the cardiovascular system [57]. In addition, the flavonoids in *D. denneaum paxt*. have also been found to have an antitumor activity [58]. In this study, metabolome analysis showed that the contents of kaempferol, quercetin, and other important flavonols in biennial *D. officinale* were significantly higher than those in three- and four-year-old *D. officinale*, and the accumulation of chalcone, naringenin, eriodictyol, dihydroquercetin, kaempferol, isoflavone, and other flavonoids in three- and four-years-old *D. huoshanense* and three-and four-years-old *D. moniliforme* was significant. As a result, the second year might be the best harvest time for *D. officinale*, while the third or fourth year might be the best harvest time for *D. huoshanense* and *D. moniliforme*.

Using transcriptomic and metabolomic data from previous studies, we screened three *Dendrobium* species with different growth years. Due to the high flavonoid content of three-year-old *D. huoshanense* and biennial *D. officinale*, we posited that the third and second years would be ideal for harvesting high-quality *D. huoshanense* and *D. officinale*. Furthermore, *D. moniliforme* had a higher medicinal and commercial value than *D. huoshanense* and *D. officinale* in terms of flavonoid content. The objective of this study was to fill the gap in the flavonoid biosynthesis pathway in three *Dendrobium* species and to provide new insights for the exploration of medicinal components and the rational selection of harvest times for these species.

## 4. Materials and Methods

### 4.1. Plant Materials and Sample Collection

Three different *Dendrobium* species (*Dendrobium huoshanense*, *Dendrobium officinale*, *Dendrobium moniliforme*) were cultivated artificially and gathered in the greenhouse of Anhui Tongjisheng Biotechnology Co., Ltd. The cultivation conditions of the samples were similar to those in our previous study [7]. Seed germination and protocorm-like bodies growth were cultured on half-strength Murashige and Skoog (MS) medium adding 6-BA 0.1 mg·L^−1^, NAA 0.5 mg·L^−1^ and 1% additives (30 g·L^−1^ sucrose + 4 g·L^−1^ agar + 20% potato) under a 12/12 h light–dark cycle (approx. 30 μmol m^−2^·S^−1^) at 25 ± 2 °C. After 6 months, the plants were transplanted into pots and placed in the greenhouse at a temperature of 25–27 °C with a light/dark cycle of 12/12 h and 60–70% relative humidity. Plant stems from one-year-old *Dendrobium huoshanense* (1Dh), *Dendrobium officinale* (1Do), *Dendrobium moniliforme* (1Dm); two-year-old *Dendrobium huoshanense* (2Dh), *Dendrobium officinale* (2Do), *Dendrobium moniliforme* (2Dm); three-year-old *Dendrobium huoshanense* (3Dh), *Dendrobium officinale* (3Do), *Dendrobium moniliforme* (3Dm); and four-year-old *Dendrobium huoshanense* (4Dh), *Dendrobium officinale* (4Do), *Dendrobium moniliforme* (4Dm) plants harvested on 10 November 2020, were used as the research object (Figure S1 (Supplementary Materials)). To extract RNA and metabolites, a portion of the materials was frozen in liquid nitrogen at −80 °C. Before being evaluated for total alkaloids and total flavonoids, the other portion was washed and dried. Furthermore, the current study included three biological replicates for all experiments.

### 4.2. Determination of Total Flavonoid and Total Alkaloid Contents

*Dendrobium* stem flavonoids were determined using the AlCl_3_ colorimetry method [59]. Using a standard graph of quercetin, the flavonoid content was determined and expressed in mg·g^−1^ of quercetin equivalents. The first step was to weigh 0.02 g of dried and crushed powder from the stem of *Dendrobium*. A solution of the powder was dissolved in 2 mL of 60% (*v*/*v*) ethanol, and the solution was then extracted through ultrasonication for two h at a temperature of 60 °C. The filtrate was centrifuged at 10,000 rpm for 10 min, and the supernatant was collected. Next, 30 mL of 5% (*w*/*w*) NaNO_2_ was added to 540 mL of the supernatant and mixed well. After 6 min, 30 mL of 10% (*w*/*w*) AlCl_3_ was added to the mixture and mixed well. After 5 min, 400 mL 1 mol/L NaOH was added to the mixture. The mixture was then left at room temperature for 15 min and the absorbance at 510 nm was measured.

Total alkaloids content was carried out according to the method described by Yuan et al. with some modifications [7]. To the powdered sample (0.1 g), 0.1 mL of ammonia water and 0.9 mL of 80% (*v*/*v*) ethanol were added. The sample and solvent were mixed thoroughly and extracted by ultrasonication for one hour. The mixture was centrifuged for 10 min at 10,000 rpm, and the supernatant was collected as the test liquid. Following that, 0.5 mL potassium hydrogen phthalate buffer solution (pH 4.5) and 0.2 mL 0.04% bromocresol green solution were added to 0.1 mL test liquid, and the mixture was kept at room temperature for 5 min. Then, 1 mL of chloroform was added to the mixture, shaken vigorously to mix the solution, and allowed to stand for 40 min at room temperature. An absorbance measurement at 416 nm was performed after aspirating 1 mL of the lower chloroform layer. Dendrobine was used as a standard to determine the total alkaloid content.

### 4.3. Widely Targeted Metabolomics Profiling

To investigate metabolite variations among different growth years and species of *Dendrobium* stem, we conducted widely targeted metabolomics studies on samples with three biological replicates for each growth year and each species. Briefly, 100 mg of fresh sample powder was dissolved in 70% methanol extraction solution, vortexed 6 times, and the samples were placed in a 4 °C refrigerator overnight. After centrifugation (12,000 rpm, 10 min), the supernatant was aspirated, and the samples were filtered through a microporous membrane (0.22 μm pore size) for UPLC-MS/MS analysis. The UPLC column was Agilent SB-C18 1.8 µm, 2.1 × 100 mm. Metware Biotechnology Ltd. was tasked with metabolite analysis on stem samples (Wuhan, China). The Analyst 1.6.3 program was used to analyze the metabolic data. Based on MWDB (Metware database), qualitative analysis of metabolites was carried out according to secondary spectral information. Metabolite quantification was performed using the multiple reaction monitoring (MRM) mode of triple quadrupole mass spectrometer. The differences between the metabolites of the two samples were maximized using OPLS-DA to find the differential metabolites (Orthogonal projections to latent structures-Discriminant Analysis). On the basis of the OPLS-DA results, the derived Variable Importance in Projection (VIP) of the OPLS-DA model for multivariate analysis was utilized to undertake a preliminary screening of differential metabolites. Foldchange ≥ 2 and foldchange ≤ −0.5, VIP ≥ 1 were the differential metabolites in our inquiry for the next step of analysis. Using the R program, we used principal component analysis (PCA) to look at the buildup of Dendrobium metabolites at different growth years. All samples were evaluated using a cluster heatmap that was developed once the data were standardized. A part of the data, including the *D. huoshanese*, *D. officinale* and *D. moniliforme* metabolomics data, were published [60,61,62].

### 4.4. RNA Extraction, Illumina Sequencing, and Analysis of DEGs

Total RNA was extracted from Dendrobium stems and evaluated for quality using the Omni Plant RNA Kit (CWBIO, Beijing, China). According to the manufacturer’s instructions, the TruSeq^TM^ RNA sample preparation kit (Illumina, San Diego, IL, USA) was utilized, and the cDNA libraries were created using mRNA from each sample. The HISAT2 software [63] was used to map the filtered reads to the reference genome (https://www.ncbi.nlm.nih.gov/genome/69090) (accessed on 5 December 2020) [4]. Gene expression was measured using Fragments Per Kilobase Million (FPKM) measurements. The DESeq2 was used to find differential expression genes, with a filter requirement of |log2(fold change)| >1 and a *p*-value of less than 0.05 [64]. TopGO and clusterProfiler 4.0 were used to enrich all DEGs in Gene Ontology (GO) and the Kyoto Encyclopedia of Genes and Genomes (KEGG) to better understand their function and critical pathways [65]. A part of the data, including the *D. huoshanese*, *D. officinale* and *D. moniliforme* transcriptome data, were published [60,61,62].

### 4.5. Gene Co-Expression Network Construction

A weighted gene co-expression network was created using the R program WGCNA packet to study genes that are strongly associated to the Dendrobium properties (years, species, and medicinal components) [66]. The soft threshold was chosen and co-expression modules were constructed using the Soft Threshold selector function to calculate the soft threshold value. The topological overlap matrix (TOM), which can reflect the similarity of the co-expression relationship between the two genes, was further developed 63 by computing the adjacent matrix on a soft threshold. Finally, to create a hierarchical clustering tree of DEGs, a hierarchical clustering approach is used. Hub genes were commonly used in the co-expression module for closely related genes with a high connection level. According to the size of the module, the top 20 genes with the strongest link were identified as hub genes. Following that, the genes in these modules were studied. We created and visualized a gene–gene interaction network using Cytoscape (v.3.6.1) [67].

### 4.6. Combination Analysis of Transcriptome and Metabolome

The DEGs and DAMs of the flavonoid biosynthesis pathway in each comparison group were examined based on the metabolite content and gene expression value in the stem of different *Dendrobium* species at different growth years. The DEGs and DAMs relevant to flavonoid production were first analyzed using pathway analysis. Furthermore, DEGs and DAMs were mapped to the KEGG pathway database to collect common pathway information in order to better understand the interaction between transcriptome and metabolome.

## Data Availability

The raw data of all RNA-Seq samples obtained in this study were deposited in the NCBI Sequence Read Archive under the project with identification number PRJNA776680 and PRJNA776418 and Genome Sequence Archive in National Genomics Data Center, China National Center for Bioinformation/Beijing Institute of Genomics, Chinese Academy of Sciences (GSA: CRA005817) that are publicly accessible at https://ngdc.cncb.ac.cn/gsa/browse/CRA005817 (accessed on 11 January 2022).

## Supplementary Materials

The following supporting information can be downloaded at: https://www.mdpi.com/article/10.3390/ijms231911980/s1.
