# Supplementary material for: A Comparison of the Flavonoid Biosynthesis Mechanisms of Dendrobium Species by Analyzing the Transcriptome and Metabolome
Source: Int J Mol Sci. 2022 Oct 9;23(19):11980. doi: 10.3390/ijms231911980 (PMC9569625; doi:10.3390/ijms231911980)
Supplement: Supplementary file 1 [file ijms-23-11980-s001.zip › ijms-1936145-supplementary.pdf]

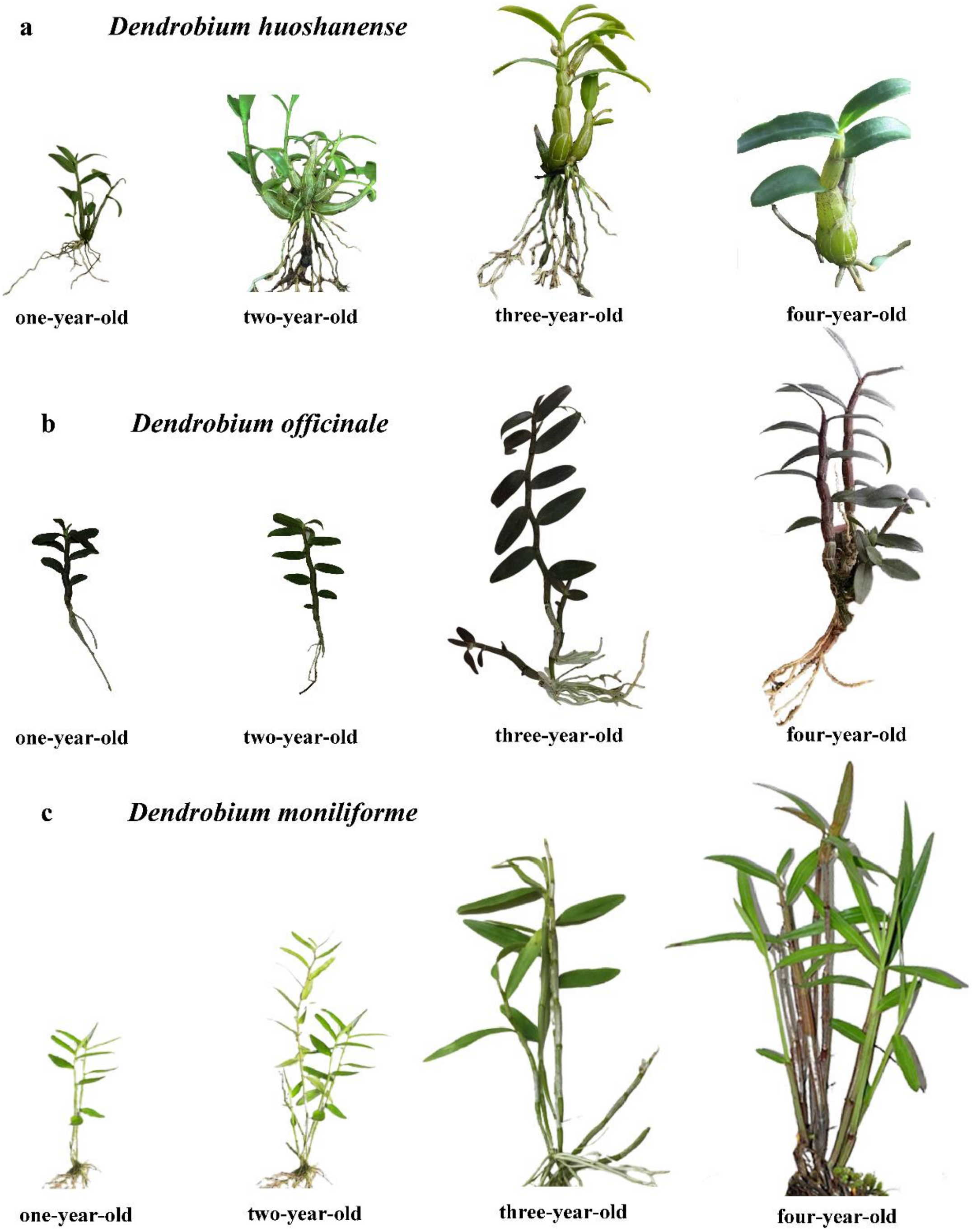

Figure S1. Plant of *Dendrobium huoshanense, Dendrobium officinale,* and *Dendrobium moniliforme* from four different years.

Table S1. Percentage of clean reads mapped to the genome for different samples

Sample	Total_Mapped	Multiple_Mapped	Uniquely_Mapped	Mapped_to_Gene	Mapped_to_Exon
1Dh_1	33064006 (78.42%)	981965 (2.97%)	32082041 (97.03%)	31120579 (97.00%)	30011321 (96.44%)
1Dh_2	32928749 (78.03%)	978800 (2.97%)	31949949 (97.03%)	31000352 (97.03%)	29921491 (96.52%)
1Dh_3	34614021 (78.03%)	1035192 (2.99%)	33578829 (97.01%)	32580676 (97.03%)	31453841 (96.54%)
1Dm_1	28214545 (74.19%)	977182 (3.46%)	27237363 (96.54%)	26444263 (97.09%)	25464796 (96.30%)
1Dm_2	28718885 (76.60%)	924803 (3.22%)	27794082 (96.78%)	26946785 (96.95%)	25850677 (95.93%)
1Dm_3	33173369 (77.94%)	1123869 (3.39%)	32049500 (96.61%)	31142523 (97.17%)	30020589 (96.40%)
1Do_1	37610002 (91.03%)	1113989 (2.96%)	36496013 (97.04%)	35151660 (96.32%)	33220556 (94.51%)
1Do_2	41944349 (90.44%)	1329779 (3.17%)	40614570 (96.83%)	39146519 (96.39%)	37214974 (95.07%)
1Do_3	40784102 (90.82%)	1246515 (3.06%)	39537587 (96.94%)	38114747 (96.40%)	36403212 (95.51%)
2Dh_1	40242160 (62.18%)	1441922 (3.58%)	38800238 (96.42%)	37716198 (97.21%)	37340258 (99.00%)
2Dh_2	46371206 (69.99%)	1273397 (2.75%)	45097809 (97.25%)	43940424 (97.43%)	42685508 (97.14%)
2Dh_3	60244250 (72.48%)	1808105 (3.00%)	58436145 (97.00%)	56946191 (97.45%)	55463209 (97.40%)
2Dm_1	65977284 (75.80%)	2137630 (3.24%)	63839654 (96.76%)	62240573 (97.50%)	60242712 (96.79%)
2Dm_2	55453115 (73.16%)	1558836 (2.81%)	53894279 (97.19%)	52542588 (97.49%)	50913032 (96.90%)
2Dm_3	62608076 (72.75%)	2167709 (3.46%)	60440367 (96.54%)	58786496 (97.26%)	56608387 (96.29%)
2Do_1	64798635 (80.36%)	2085070 (3.22%)	62713565 (96.78%)	59835030 (95.41%)	57401516 (95.93%)
2Do_2	68113658 (87.89%)	1925995 (2.83%)	66187663 (97.17%)	63993631 (96.69%)	61817781 (96.60%)
2Do_3	71939597 (86.74%)	1856145 (2.58%)	70083452 (97.42%)	67935641 (96.94%)	65116079 (95.85%)
3Dh_1	35197051 (77.44%)	1080708 (3.07%)	34116343 (96.93%)	33144567 (97.15%)	32236933 (97.26%)
3Dh_2	30996592 (68.71%)	947484 (3.06%)	30049108 (96.94%)	29078432 (96.77%)	27960570 (96.16%)
3Dh_3	35851401 (74.46%)	1107761 (3.09%)	34743640 (96.91%)	33733054 (97.09%)	32936804 (97.64%)
3Dm_1	26944430 (69.72%)	890061 (3.30%)	26054369 (96.70%)	25116040 (96.40%)	24078787 (95.87%)
3Dm_2	29548148 (69.36%)	1013033 (3.43%)	28535115 (96.57%)	27500030 (96.37%)	26319615 (95.71%)
3Dm_3	25671913 (68.21%)	881071 (3.43%)	24790842 (96.57%)	23914898 (96.47%)	23019254 (96.25%)

3Do_1	40773054 (87.97%)	1190356 (2.92%)	39582698 (97.08%)	38095898 (96.24%)	36064514 (94.67%)
3Do_2	41847425 (89.53%)	1212063 (2.90%)	40635362 (97.10%)	39294823 (96.70%)	37383957 (95.14%)
3Do_3	41266606 (88.97%)	1156989 (2.80%)	40109617 (97.20%)	38638322 (96.33%)	36757569 (95.13%)
4Dh_1	29855494 (64.69%)	867651 (2.91%)	28987843 (97.09%)	28093649 (96.92%)	27012095 (96.15%)
4Dh_2	35557040 (69.80%)	1010392 (2.84%)	34546648 (97.16%)	33496930 (96.96%)	32264361 (96.32%)
4Dh_3	32825375 (67.57%)	976359 (2.97%)	31849016 (97.03%)	30897057 (97.01%)	29815147 (96.50%)
4Dm_1	26146092 (68.12%)	1024542 (3.92%)	25121550 (96.08%)	24227961 (96.44%)	23198038 (95.75%)
4Dm_2	24395296 (68.21%)	892917 (3.66%)	23502379 (96.34%)	22645430 (96.35%)	21597043 (95.37%)
4Dm_3	25465146 (64.69%)	842401 (3.31%)	24622745 (96.69%)	23714741 (96.31%)	22668871 (95.59%)
4Do_1	40347591 (78.51%)	1320129 (3.27%)	39027462 (96.73%)	37254277 (95.46%)	35080164 (94.16%)
4Do_2	41108594 (84.71%)	1327035 (3.23%)	39781559 (96.77%)	38115459 (95.81%)	35797246 (93.92%)
4Do_3	34379639 (77.41%)	1200928 (3.49%)	33178711 (96.51%)	31741486 (95.67%)	29767381 (93.78%)
